# On the road to personalised and precision geomedicine: medical geology and a renewed call for interdisciplinarity

**DOI:** 10.1186/s12942-016-0033-0

**Published:** 2016-01-28

**Authors:** Maged N. Kamel Boulos, Jennifer Le Blond

**Affiliations:** The Alexander Graham Bell Centre for Digital Health, University of the Highlands and Islands, Elgin, IV30 1JJ Scotland, UK; Department of Earth Science and Engineering, Imperial College London, South Kensington, London, SW7 2AZ England, UK

**Keywords:** Medical geology, Geomedicine, Precision medicine

## Abstract

Our health depends on where we currently live, as well as on where we have lived in the past and for how long in each place. An individual’s place history is particularly relevant in conditions with long latency between exposures and clinical manifestations, as is the case in many types of cancer and chronic conditions. A patient’s geographic history should routinely be considered by physicians when diagnosing and treating individual patients. It can provide useful contextual environmental information (and the corresponding health risks) about the patient, and should thus form an essential part of every electronic patient/health record. Medical geology investigations, in their attempt to document the complex relationships between the environment and human health, typically involve a multitude of disciplines and expertise. Arguably, the spatial component is the one factor that ties in all these disciplines together in medical geology studies. In a general sense, epidemiology, statistical genetics, geoscience, geomedical engineering and public and environmental health informatics tend to study data in terms of populations, whereas medicine (including personalised and precision geomedicine, and lifestyle medicine), genetics, genomics, toxicology and biomedical/health informatics more likely work on individuals or some individual mechanism describing disease. This article introduces with examples the core concepts of medical geology and geomedicine. The ultimate goals of prediction, prevention and personalised treatment in the case of geology-dependent disease can only be realised through an intensive multiple-disciplinary approach, where the various relevant disciplines collaborate together and complement each other in additive (multidisciplinary), interactive (interdisciplinary) and holistic (transdisciplinary and cross-disciplinary) manners.

## Introduction: medical geology—a re-emerging approach

The concept of medical geology is not new. The study of the relationship between the environment and health, and the fundamental recognition of disease as a consequence of environmental exposure, was noted as far back as Hippocrates (ca. 460–370 BC), the Greek Physician referred to as the ‘Father of Medicine’. However, whilst the term medical geology is not novel, the capacity for its use in exploring previously unanswered health issues is receiving more attention and is helping to bring together a multitude of scientific disciplines to solve fundamental questions within public health.

Medical geology can be defined broadly as the study of the interaction between the environment and health. More specific descriptions aim to categorise the ‘environment’ portion of the term and state that medical geology is the study of health problems caused by, or exacerbated by, geologic materials and processes (e.g., [1]), giving examples such as the exposure to various trace elements in waters/rocks/soils or to volcanic ash/gas or dust storms. Interestingly, it is both the presence and absence of constituents within the environment that can be causal agents in the occurrence of disease. Although many definitions declare that the geological features must be ‘naturally occurring’, a rigorous investigation in medical geology cannot afford to disregard the impact of quasi-natural or anthropogenic (human-made) sources in the environment.

Medical geology studies frequently draw parallels with those carried out under the guise of occupational health, but they are generally differentiated by considering where the exposure occurred—during employment or not. Further to this, many of the methods of investigation, result interpretation and regulatory implications currently employed in medical geology were initially developed by occupational health specialists, who strived to set limits and standards to reduce employees’ exposure to hazardous conditions in various industrial occupations. However, segregating occupational and environmental health will grossly underestimate exposure, and so both must be considered together to give a representative estimation.

As part of a comprehensive study, full consideration must be given to develop a wider understanding of how both natural and anthropogenic features act (and interact) to modify the environment, and how occupational and environmental exposures contribute to disease occurrence. To explore and understand the complex relationships between the environment and health, a range of disciplines must be engaged to bring expertise on discrete portions of the project. In addition to the complexities of each individual science, the challenges of inter-disciplinary communication and differing working practices must be overcome to enable researchers to formulate and test hypotheses, and ultimately draw conclusions and remediation recommendations. Medical geology is truly an interdisciplinary science, and the approach advocated in medical geology studies is gaining support in communities wishing to address key health issues around the world.

## Exposure in the environment

The human body is well adapted to cope with living on the Earth’s surface. There are, however, scenarios where the natural processes that occur to shape the physical landscape create a less habitable environment, either for humans or other living organisms. Humans perhaps first observed this anecdotally through local, experience-based knowledge, without understanding empirically what they were seeing. But now many of these scenarios can be investigated scientifically to identify which constituents within the environment are potentially contributing to poor health.

It is important to acknowledge that the environment is complex, and attempt to capture every concurrently occurring process will be beyond the scope of many studies. It is, however, useful to identify the biome(s) within which the potential components that could impact health reside, namely the atmosphere, biosphere, hydrosphere and lithosphere. Biomes should not be considered discrete, and interaction between the biomes is universally evident; for example, the weathering and breakdown of parent rock over time to form soil, which can be broken down further and taken up by fauna and flora or lifted into the atmosphere through aeolian (wind) action. Further to this, chemical weathering acts on the rock via alteration and degradation, an output of which can be the migration of constituents originally hosted in the rock into waters (soil water, running and stagnant water bodies, for example) either fully dissolved into solution or in suspension.

**Fig. 1:**
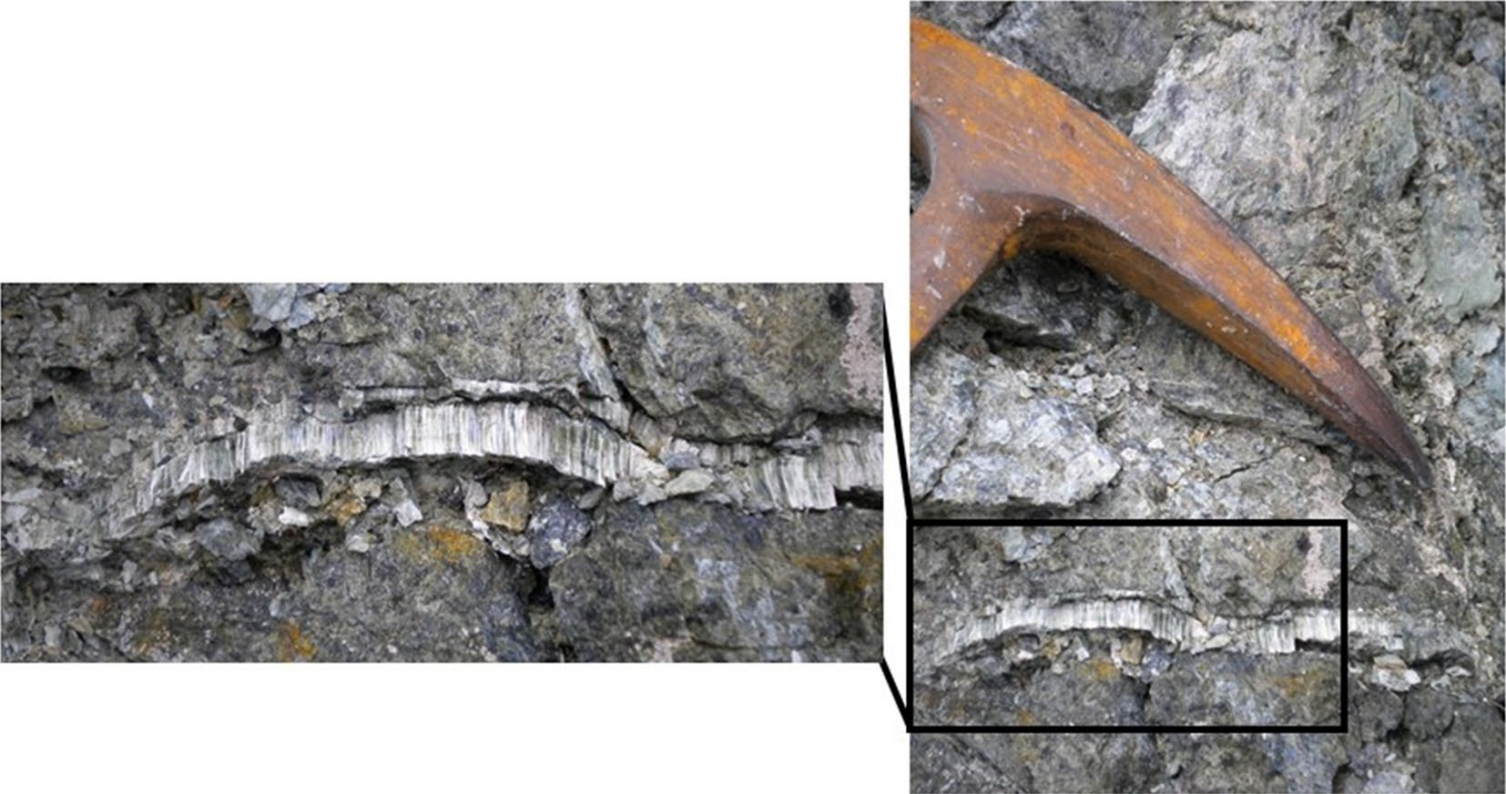
Antigorite asbestos veins in host serpentinite rocks in New Caledonia. The geological hammer is included for scale

Medical geology studies will typically differentiate these environmental components, linked with disease, to be either inorganic or organic. For example, dust (particulate matter) can be composed of biologically-derived matter from animal or microbial origin and contain fungi/bacteria. Inorganic forms of particulate matter can be minerals, such as asbestos or silica, or a suite of minerals (quartz, plagioclase, pyroxene, etc.) that can be found in a range of geological deposits, such as volcanic ash or sedimentary rock. A plethora of information exist in occupational health literature on particulate matter exposure in occupational settings, mainly due to the fact that both the component in question (i.e., the form/type of particulate matter) and the exposure can be well characterised. There are fewer studies detailing the natural environment, which is complicated by the fact that particulate matter in environmental exposure studies is often composed of a large array of constituents, making it difficult to fully capture the exposure. Take, for example, asbestos, which describes a group of minerals that are heat and corrosion resistant and were used extensively as insulation, building materials and in friction products (such as brakes), and has subsequently been shown to cause mesothelioma in humans after exposure by inhalation. Asbestos minerals occur naturally in the environment, and one such an asbestos mineral form—antigorite—can be found in New Caledonia (an overseas territory of France located in the southwest Pacific Ocean) in the native serpentinite rocks (Fig. 1). This rock is crushed and used in road construction, and has been associated with malignant mesothelioma in local populations (e.g., [2]).

The presence or absence of elements, the constituents of minerals, in the environment can also be associated with disease. Consider the example of arsenic (As), a metalloid and known carcinogen [3] that is ubiquitous in the environment and can originate from both natural and anthropogenic sources. Arsenic is relatively common in some sulphide deposits (such as those containing pyrite, galena and chalcopyrite), and the weathering of the primary mineral in these rocks may release As, the form of which is highly dependent on the conditions within the environment (hydrogen ion activity—pH, redox potential—Eh, temperature, and presence of microbes, organic matter or clay minerals, for example). Elevated As has also been measured in soils and waters affected by natural geothermal activity (e.g., [4]) and anthropogenic activities (such as the mining and burning of As-laden coal; e.g., [5]).

Conceptualising biomes enables researchers to hypothesise possible migration routes of components within the environment, in combination with information on the form and mobility, ultimately allowing investigators to trace the origin (source). However, the exposure pathway must not only consider the release, fate and distribution of the component within the environment, but also the route by which populations are exposed. While they will vary for different organisms, it is typical to consider dermal, inhalation and ingestion as viable routes of exposure. The ingestion of elevated arsenic levels in drinking water and diet (mainly through seafood [6]) has been a widely recognised issue, linked with an assortment of health problems, such as skin and bladder cancer, and other non-carcinogenic effects such as skin lesions, neurological and hepatic effects (e.g., [7]). Other routes of arsenic exposure, however, can include inhalation of particulate matter from As-bearing soils, industrial emissions and wastes, as well as the ingestion of As-rich soils in food.

## The balance between essential and toxic elements

**Fig. 2:**
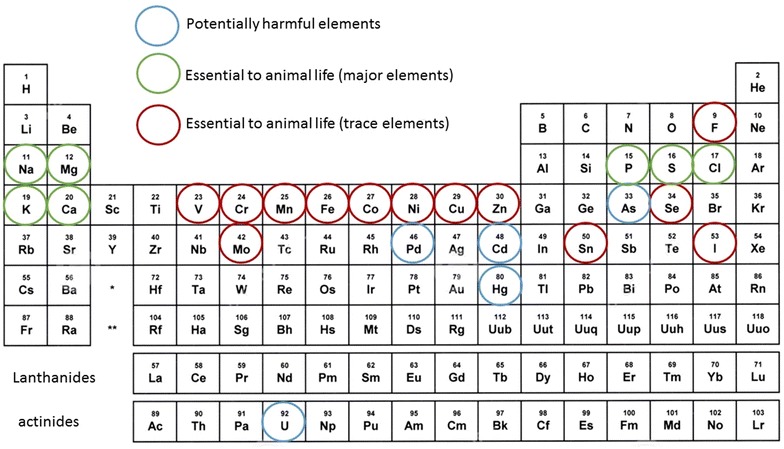
Periodic table noting the elements that are potentially harmful and those that are essential to humans (data from [8])

We introduced the idea of chemical elements as ‘contaminants’ (either natural or anthropogenic) in the environment; however, some of these elements are known to be essential to higher animals/humans (Fig. 2). These essential elements are assimilated in the body via our diet and the air we breathe. The requirements for most of life functions are met by ingestion of plant material, soil (accidental or intentional) and in water. Examples of areas where animals/humans have sought out naturally occurring mineral deposits (such as salts) to supplement dietary intake are evident in the environment. When referring to elevated or depleted levels of elements within the environment, it is commonly assumed that these levels are above or below (respectively) average or background values, which can be a consequence of natural processes in the environment or anthropogenic inputs.

‘Trace’ elements are of particular interest in medical geology, so called because they describe an array of elements (Fig. 2) found at very low (mg kg^−1^) concentrations in the natural environment. In general, deficiencies in these trace elements will lead to health disorders, but correspondingly an excess may also result in health problems. Hence, medical geology must attune to the idea that either deficiency or excess of components within the environment may be a causal factor in disease. In humans, selenium (Se), fluorine (F) and molybdenum (Mo) are examples of trace elements where the concentration range that determines whether the dose is essential or harmful to human health (termed the ‘therapeutic range’) is only in the range of µg L^−1^. Deficiencies in Se in humans, for example, was found to be linked with increased risk of mortality, poor immune functioning and cognitive decline, whereas Se supplementation has been shown to be important for successful reproduction in males and females, to reduce the risk of autoimmune thyroid disease and to have some antiviral effects [9]. Over supplementing the diet with Se when the recommended daily dose is already achieved through dietary intake is not recommended as this can have adverse health effects.

## Chemical speciation and mobility

The potential for an element (compound or mineral in the broader sense) to cause health problems, depends not only on the absence or presence of the element, but also the speciation. The speciation of an element will control the mobility and distribution in the environment, and the surface charge will likely have a bearing on the toxicity within the living organism. Extensive kinetic and thermodynamic modelling, coupled with laboratory experiments and study of the biological interaction with elements, has progressed our understanding regarding the form of many elements in the environment, and enabled researchers to predict exposure pathways from source contamination events, for example.

As previously stated, environmental conditions have a dominant influence on elemental speciation, which determines how soluble the element is and therefore dictates how mobile it may be in the environment. Temperature, presence of other cations or anions (positively and negatively charged ions, respectively), biological processes, surface reactivity and presence of organic matter are important controls on element speciation, but pH and Eh are the leading factors governing solubility. In general terms, in high pH anions such as As, Mo, P (phosphorus), Se, Te (tellurium) and B (boron) are relatively more mobile than most cations such as Cd (cadmium), Cu (copper), Hg (mercury) and Pb (lead) (Table 1), and the presence of organic matter and clay minerals can act as sorption surfaces to ‘fix’ elements within biomes such as the soil [10].

**Table 1:** Generalised relationships between the environmental conditions, as categorised by pH and Eh, and the mobility of elements (data from [11])

Relative mobility of element	Environmental condition			
	Oxidising	Acidic (low pH)	Neutral (pH 7)—alkaline (high pH)	Reducing
Very high	I	I	I, Mo, U, Se	I
High	F, Mo, U, Ra, Se, Zn	Co, Cu, F, Hg, Mo, Ni, U, Ra, Se, Zn	F, Ra	F, Ra
Moderate	As, Cd, Co, Cu, Hg, Ni	As, Cd	As, Cd	
Low	Be, Bi, Pb, Sb, Tl	Be, Bi, Fe, Mn, Pb, Sb, Tl	Be, Bi, Fe, Mn, Pb, Sb, Tl	
Very low (immobile)	Al, Cr, Fe, Mn	Al, Cr	Al, Co, Cr, Cu, Hg, Ni, Zn	Al, As, Be, BiCd, Co, Cr, Cu, Hg, Mo, Ni, Pb, U, Sb Se, Tl Zn

For the full names of the elements/element symbols in this table, please refer to [12]

Environmental conditions, known to influence elemental speciation, are moderated by processes occurring with and between the biomes. Considering the lithosphere, the nature and properties of the soil are strongly influenced by the parent rock, and other soil forming processes that are determined by the climate, topography of the land, presence (or absence) of vegetation and management practices (such as ploughing and fertilising). Lateritic soils formed in tropical (hot and wet) regions have well defined soil horizons that have been heavily leached over time, with resulting characteristic layerings in the soil profile that are either enriched or depleted in minerals/elements. Within the lateritic profile layers, metals such as Co (cobalt), Cr (chromium), Mn (manganese) and Ni (nickel) are enriched and can be extracted by mining, which has historically been considered an occupational hazard. As a demonstration of medical geology preceded by anecdotal evidence, the words ‘kobalt’ and ‘nickel’ in German denote a goblin and a scamp, thought to be evil spirits that haunt the mines and cause harm, and in the seventeenth century, German church services offered prayer to protect the miners [13]. Mining and other events that act to perturb the natural environment (including natural hazards such as earthquakes), expose previously contained deposits that may initially be less stable and more reactive due to the sudden change in environmental conditions. Open cast mines, if not adequately remediated, pose a risk to surrounding populations from exposure to airborne particulate matter from the mine, or from interaction between the exposed deposits and local water sources. While layers in the sub-strata are enriched, the surface soils in laterite profiles are strongly depleted in most essential elements or those elements are present but ‘immobilised’ in mineral complexes such as iron-manganese hydroxides. Although laterites are considered an extreme form of weathering, low concentrations of Co in leached soils around the world are a common cause of health problems for ruminants (appetite loss, wasting and death), as Co is essential for the production of vitamin B12 (livestock health is important in human food security).

The soil properties will, therefore, influence the trace element concentration in the flora and fauna that residue on it, depending on the propensity for each organism to take up elements. Elements such as Al (aluminium), Cr and Ti (titanium) are relatively poorly assimilated by plants, whereas others such as Cd, Co, Mo and Se can readily enter plants to become part of the food chain [14]. On the lateritic soils of New Caledonia, there is an abundance of endemic ‘metallophyte’ plants, which have the ability to survive on the metal-rich lateritic soils, and indeed the distribution of these plants has been employed to locate metal-rich outcrops, and metallophytes have been advocated as potential tools for remediation where metal concentrations are elevated (e.g., mine tailing sites). The concentration of bioaccessible element, that is the amount of element that can be incorporated into the plant (i.e., crosses the cellular membrane) from the soil, typically either in a free ionic form or as complexes, can be modulated in some species. Some plants have been known to adapt to changing element concentrations. These plants have mechanisms in place to modify intake in situations of deficiency (i.e., by altering the pH of the soil water near the roots of the plant) and in some cases exclude elements where external concentrations are in excess. In terms of food crops, the concentration of trace elements in leguminous crops, pulses and cruciferous crops will vary correspondingly with the soil type, whereas cereal grains are less likely to be impacted by soil trace element concentration (the exception to this rule is Se and Zn (zinc) that do tend to be reflected in the cereal grains) [15].

## Bioavailability of elements and disease onset

Lack of certain element(s) within the soil can directly affect the flora and fauna living in and on it, and incorporation of excessive levels of elements into food items can potentially elevate levels in the human diet. Nevertheless, it is not only the bioaccessibility of the element that is important but also the bioavailability. In humans, bioaccessibility is the fraction that is incorporated into the body (e.g., into the gastrointestinal tract or respiratory system), whereas bioavailability describes the fraction of the dose that reaches the body’s systemic circulation (blood stream). While geochemical surveys can be used to determine the concentration and distribution of elements in various compartments of the environment (such as soil, water and air), and by comparison with other surveys to identify whether the levels are above or below that of the background, bioaccessibility and bioavailability studies are less well defined. However, if the contaminant concentrations found in the environment are used to judge exposure, then there is the potential to overestimate exposure as a portion of this contaminant will likely not be bioaccessible and/or bioavailable. This also raises the issue of the importance of being able to recognise potential differences in correlation and causation.

Speciation can be a strong indicator of an element’s potential bioaccessibility and bioavailability, and is particularly true of metals and metalloids. Arsenic, for example, exists in four main valence states; 0 (elemental), 3, 5 and −3 (a gas), and is found in the environment as inorganic and organic compounds. The trivalent form of arsenic [As(III)] is more stable in reducing environments, and the pentavalent form [As(V)] in oxidising environments. It has been shown that biologically, inorganic As is ~100 times more toxic than organic As compounds [16], and the As(III) is ~60 times more toxic than As(V) [17]. There is also evidence to suggest that bioavailability can vary between different populations (for example adults vs. children) and species. Aluminium, for example, is one of the most abundant minerals on Earth, as both inert and bioavailable forms. Certain species of fish and plants are particularly susceptible, and even trace amounts can cause adverse physiological effects [18]. Bioavailability is also influenced by dietary factors; for example, it has been shown that the absorption of Pb via the gastrointestinal tract of children can be substantially reduced if the diet is relatively high in calcium (e.g., [19]).

In addition, it is crucial to investigate the potential mechanisms behind disease initiation or exacerbation to help explain correlations between disease and environmental contaminant exposures. The physiopathology and progression of the disease is often complex, but more simplistic mechanisms of toxicity can be explored using toxicology assays. In vitro experiments can be used to assess the impacts from exposure at the cellular level to ascertain bioreactivity, but only in vivo testing (or clinical trials) can assess the bioavailability. Nonetheless, caution must be taken when extrapolating the results from in vivo testing (i.e., animal models) to humans, as it is well known that the animal and human responses are not always consistent. Improving on the conventional sequential leach tests, in vitro assays have been developed in an attempt to replicate the conditions within the body, where the component crosses into the blood system. Simulated fluids, such as gastric fluids or phagolysosomal fluids, can be used in assays to determine the solubility of elements or compounds (after ingestion and inhalation, respectively), allowing the estimation of potential bioavailability once in the body.

Basic, low cost in vitro assays can be employed as a ‘first test’ of potential toxicity, and have been used in situations where information about a sample is needed rapidly to inform recommended levels of exposure. For example, in cases where populations are exposed to relatively large concentrations of volcanic ash from recent volcanic activity, it is important to know what the ash is composed of in terms of its mineralogy and chemical composition (predominantly controlled by the geological setting), in combination with the concentration of particles (and their distribution in space and time) and the physical characteristics such as the size and shape. However, before any large-scale investigation, it is often worthwhile to carry out initial analyses on a few representative samples and screen the ash for components that are known to be hazardous to health. During the eruption of Rabaul volcano in Papua New Guinea in 2007, a rapid assessment of the potential health hazard found that the ash did not contain cristobalite, a form of crystalline silica (a group of minerals associated with respiratory health problems), and did not elicit reactivity during in vitro assays to test for oxidative capacity (using ascorbic acid to monitor oxidative stress) and erythrolysis (to measure red blood cell death) [20].

## Designing a study

In medical geology, as with many health studies, the primary aims are to identify and find the cause of disease, to enable intervention and ultimately prevention. This promotes the need for a thorough investigation into the problem, to make sure that features within the exposure pathway are not misinterpreted or overlooked.

**Fig. 3:**
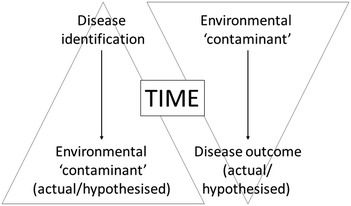
Schematic representation of how medical geology studies are typically initiated, with either disease or environmental contaminant

There are typically two fundamental approaches that initiate an investigation in medical geology, namely (1) the identification of a disease (or poor health) in persons or populations, and (2) the recognition of the presence of an element, mineral or compound in the environment that has the potential to cause harm to health (Fig. 3). The latter (second) approach can be applied when speculating health outcomes after contamination events (such as pollutant release) and natural hazards, when there is a sudden perturbation in the environment. The factor of time (temporal dimension) is fundamental in health studies, where thought must be given not just to all of the links that create the pathway from environment to disease, but also the influence of time on the formation of the contaminant in the environment, on the exposure (i.e., chronic or acute) and on disease initiation (i.e., latency). GIS (geographic information systems) methods can be of great help in the spatio-temporal modelling and visualisation of environmental contaminants and their spread over time and space (e.g., [21]).

In the former (first) approach, once a disease has been identified, carefully orchestrated epidemiological studies can help constrain the incidence and distribution. Data can be collected as primary or secondary data (the latter type describing the use of data originally collected for an alternative purpose). The use of cohort and cross-sectional studies instead of aggregated population data often proves to be valuable in capturing individual exposure. In addition, by fully characterising the nature of the disease, this may allude to the causal agents, as already known and documented associations may exist between elements or metals, for example, and target organs (e.g., Cd is known as a nephrotoxic agent that impacts the kidneys, while Hg can affect brain and heart functioning). One advantage of a more detailed epidemiological investigation into the disease is the capacity for identifying confounding factors such as disease susceptibility due to physiological and potentially even genetic variations. Defining exposure is perhaps one of the most challenging aspects of any medical geology investigation, as exposure may occur over long periods of time—days, years and even decades. Further to this, exposure to the causal agent may have been at some point in the past, which is the case for asbestos exposure and mesothelioma, where symptoms manifest often several decades after first exposure.

In the second approach, if an environmental survey (either routine or exploratory) identifies the presence of a certain element or compound that could potentially be a hazard to human health, then an extensive investigation can be undertaken to map the distribution of the feature within the domain of interest. Field data and data sourced from other methods (such as remote sensing) are commonly used to compile sets of variables that aim to describe the domain of interest. However, the aim or aims of sampling the environment must be set at the beginning of the investigation to enable a suitable sampling protocol to be developed and ensure that the correct statistical analysis, of that data once collected, can be carried out (e.g., [22]). Much of the practical fieldwork in small-scale medical geology studies is limited by project finance, and perhaps the most significant drain on this resource is sample analysis, which will often influence the number and type of samples that can be collected. Physical samples, such as soils, water, rock and particulate matter collected onto filters, must be collected in a way that is conscious of subsequent analysis. For example, if the concentration of Zn is to be determined by inductively coupled plasma-atomic emission spectroscopy (ICP-AES) in the particulate matter collected from forest fires, the collection methods (passive or active) and sampling duration must ensure that enough material is retrievable from the filters to fit within the operating calibration range and detection limits of the instrument. Likewise, if pesticides are being measured in waters on agricultural land, the appropriate sampling vessels must be used and correct storage/transport conditions must ensure that the components within the sample do not breakdown or alter into products of the pesticide.

## Geomedical engineering and the conventional geoinformatics aspects of medical geology

‘Geomedical engineering’ is a term first coined in 2009 by Ur-Rehman [23] in an article entitled “Geomedical engineering: a new and captivating prospect” to describe the discipline of applying engineering practice principles to medical geology issues for the purposes of preventing or mitigating the geological, geochemical and geoenvironmental hazards and risks to human and animal health. Medical geologists and geomedical engineers work very closely with each other. Medical geologists are concerned with examining the roles played by soils, rocks and groundwater (drinking water travels through the rocks and soils as part of the hydrological cycle) in human and animal health, documenting the important influences of the geoenvironment on the geographical distribution of various diseases and health problems, as well as the changes that occur in those influences over time and space, in order to devise adequate programmes for controlling harmful exposures in risky environments. Geomedical engineers provide medical geologists with the necessary engineering techniques and solutions (digital/ICT—information and communication technologies, and other technologies) to execute (1) geomedical hazard identification and quantification; (2) rock, soil and water treatment in order to prevent certain diseases; and (3) the measures to correct the deficiency and control the toxicity of relevant elements in natural environments. Specifically, geomedical engineering deals with the following methods and techniques:

- Geomedical hazard evaluation and quantification techniques: to evaluate the geometry and extent of hazardous regions and Earth processes that pose risks to human health, and to establish the relationships between diseases and the deficiencies or excesses of certain elements in these regions (e.g., goitre/hypothyroidism and iodine deficiency);
- Water treatment and quality improvement techniques: to control or prevent known health risks by adding or reducing certain elements and substances in drinking water;
- Geomedical upgradation techniques of Earth materials: to identify and correct deficiencies of relevant elements in the soil, rock or groundwater of certain areas; and
- Geomedical reclamation techniques of Earth materials: to identify and rectify excesses of toxic elements in soil, rock or groundwater of a region [23].

Armed with geoinformatics methods and software tools, including GIS, geomedical engineers, in consultation with medical geologists, prepare maps of areas of interest to visualise the extent and toxicity of health-hazardous elements in Earth materials and waters, marking currently unsafe regions and their risk levels, as well as any regions that are likely to become risky at some point in the future (e.g., [21]). Geomedical engineers, again working hand in hand with medical geologists and public health/clinical specialists, would then advise on, and guide, the implementation of any necessary preventive or corrective measures by running appropriate soil and water treatment and management programmes to control toxicity risks, and to support a sustainable use of natural resources and a healthy balance between humans and their geoenvironment, e.g., reclamation techniques by artificial addition of certain minerals or salts to Earth materials and water, or techniques to stop the artificial addition of toxic substances from external (e.g., industrial) sources to the environment [23].

## From population health to individual person’s health: personalised and precision geomedicine

Geomedicine targets the health and clinical management of *individual* persons/patients rather than of whole populations. As such, geomedicine can make an important contribution to the delivery of personalised and precision medicine (PPM) [24, 25]. Both population and the individual person’s health are interlinked and inseparable in many ways, but ‘population health’ has traditionally been the focus of public health geoinformatics. Now this is gradually changing, with geoinformatics methods and GIS software tools also playing an important role in the delivery of geomedicine (focused on individuals), besides their more traditional role in spatial epidemiology (focused on populations).

Our health depends on where we currently live, as well as on where we have lived in the past and for how long in each place (place history). Place history is particularly relevant in conditions with long latency between exposures and clinical manifestations, as is the case in many types of cancer and chronic conditions. Indeed, Davenhall [26–28] rightly suggests that a patient’s geographic (or place) history should routinely be considered by physicians when diagnosing and treating individual patients. It provides useful contextual environmental information (and the corresponding health risks) about the patient, and should ideally form (together with genetic/genomic information) an essential and routine part of every electronic patient/health record [29–31], if we were to realise the full vision of PPM.

Wild [32] proposed the concept of the ‘exposome’, representing all non-genetic environmental exposures (including lifestyle factors) from conception onward, to complement the ‘genome’ when studying disease aetiology. Exposomics is the discipline concerned with the analysis of exposure to, and interplay of, all environmental stressors from internal (own body, e.g., inflammation, lipid peroxidation and gut bacteria) and external sources, including chemical/geological factors, to help us better understand, manage and prevent chronic disease development [33, 34].

The ‘My Place History’ app (ESRI, Redlands, CA, USA) for the iPhone and iPad [35] offers a good, albeit incomplete, demonstration of the concepts and potentials of geomedicine and place history, serving as a ‘proof of concept’ of this emerging domain. My Place History is described by its publisher as “a way to link public health information with personal environmental experience (…) bringing everyday information into the physician/patient relationship (…) to achieve a greater understanding of how local environments affect health”.

**Fig. 4:**
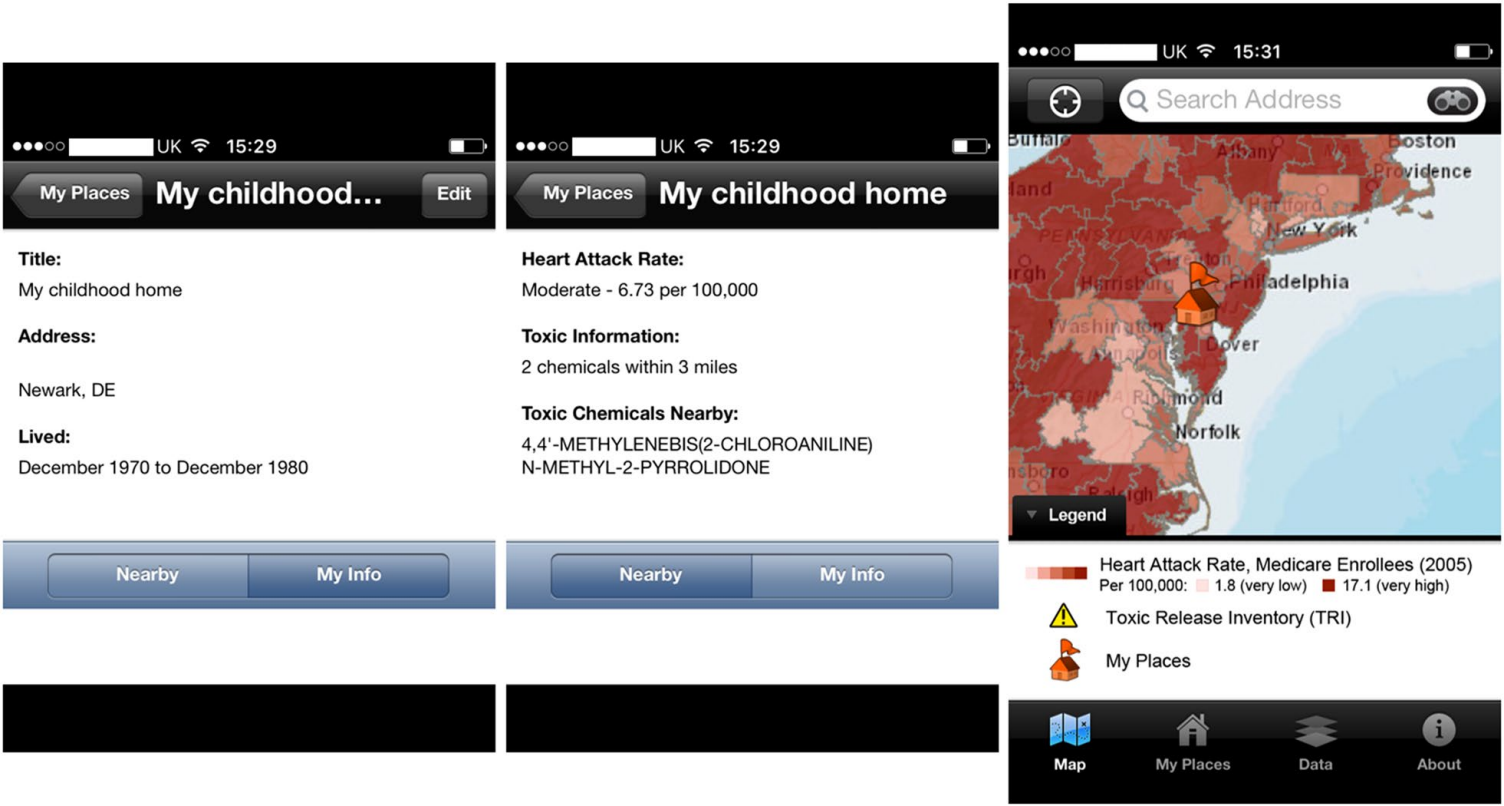
Screenshots of My Place History iPhone app version 1.4 (ESRI, Redlands, CA, USA) showing a user defined place (‘My Childhood Home’) and the health risks associated with it presented as text and on a zoomable map. Toxic chemicals are marked where they occur on the map using *yellow warning triangle signs* (none is shown in this figure)

The app (latest version at time of writing was version 1.4, dated 24 May 2012) is currently limited to places in the United States and is not integrated with any form of electronic health/patient records. It allows users (either clinicians, checking places reported by their patients, or laypersons/patients) to input and check the different current and past places they lived or worked at (i.e., place history) for possible health risks (Fig. 4).

The app links personal place history (US street addresses) to several governmental databases in the public domain, including the 2008 version of the Toxic Release Inventory (TRI) [36] of the US Environmental Protection Agency, the US National Library of Medicine’s TOXMAP and Haz-Map (chemical effects on human health) [37, 38], and the US Centers for Medicare and Medicaid Services (CMS) [39] and Dartmouth Atlas Project’s [40] heart attack rates (acute myocardial infarction) per 100,000 Medicare enrolees (2005). The app enables users to link and gather general information about the distance or proximity to certain specific events, hazards or exposures, and thus learn about how healthy or unhealthy a place they live/lived or work/worked at is/was. By engaging the layperson/patient and offering them self-discoverable insights and information about their own health and health risks, the app integrates some element of ‘participatory’ medicine, a key aspect of P4 (systems) medicine (predictive, preventive, personalised and participatory medicine) [41] or P5 (if we add ‘precision’) medicine.

However, My Place History leaves much to be desired. The authors wish to see a future version of the app covering more countries besides the United States, and offering a standardised interface for establishing electronic patient/health record links. Adding support for Android and Windows 10 operating systems would be highly desirable. More medical geology and other relevant public data sources could and should be integrated (and regularly updated in the app) to enable the compilation of a more complete picture of the various health risks at a given location. Live and historical data should be included, so that changes in near real time (if relevant) and over multiple past periods of time are covered by the various datasets and correctly mapped to the date range a user has lived at a given place (risks can vary at the same place over time). It should also be useful if risks could be quantified in some way according to the duration spent at a given place and whether there were any breaks during that period. Explanations should be given in layperson’s language about the nature, meaning and health implications of any toxic chemicals found in locations on the user’s place history list (rather than just listing the names of those chemicals in the current app version). Integration with the user’s location history on today’s smartphones equipped with global positioning system (GPS) and other geolocation technologies can be helpful, if user’s location privacy can be maintained. Users can even use the app to check for places they are about to travel or move to, and decide if they wish to visit or live at those places in the context of their personal health/medical profile. ESRI states (at [35]) that future versions of My Place History will include additional databases, such as water quality (e.g., levels of perchlorate contamination in the water supply; perchlorate is a chemical that has been associated with thyroid disease and cancer [28]), lead contamination, cancer, mortality, school performance, crime and poverty.

## Grafting disciplines in modern medical geology: the needs for interdisciplinarity and a well-coordinated holistic approach

**Fig. 5:**
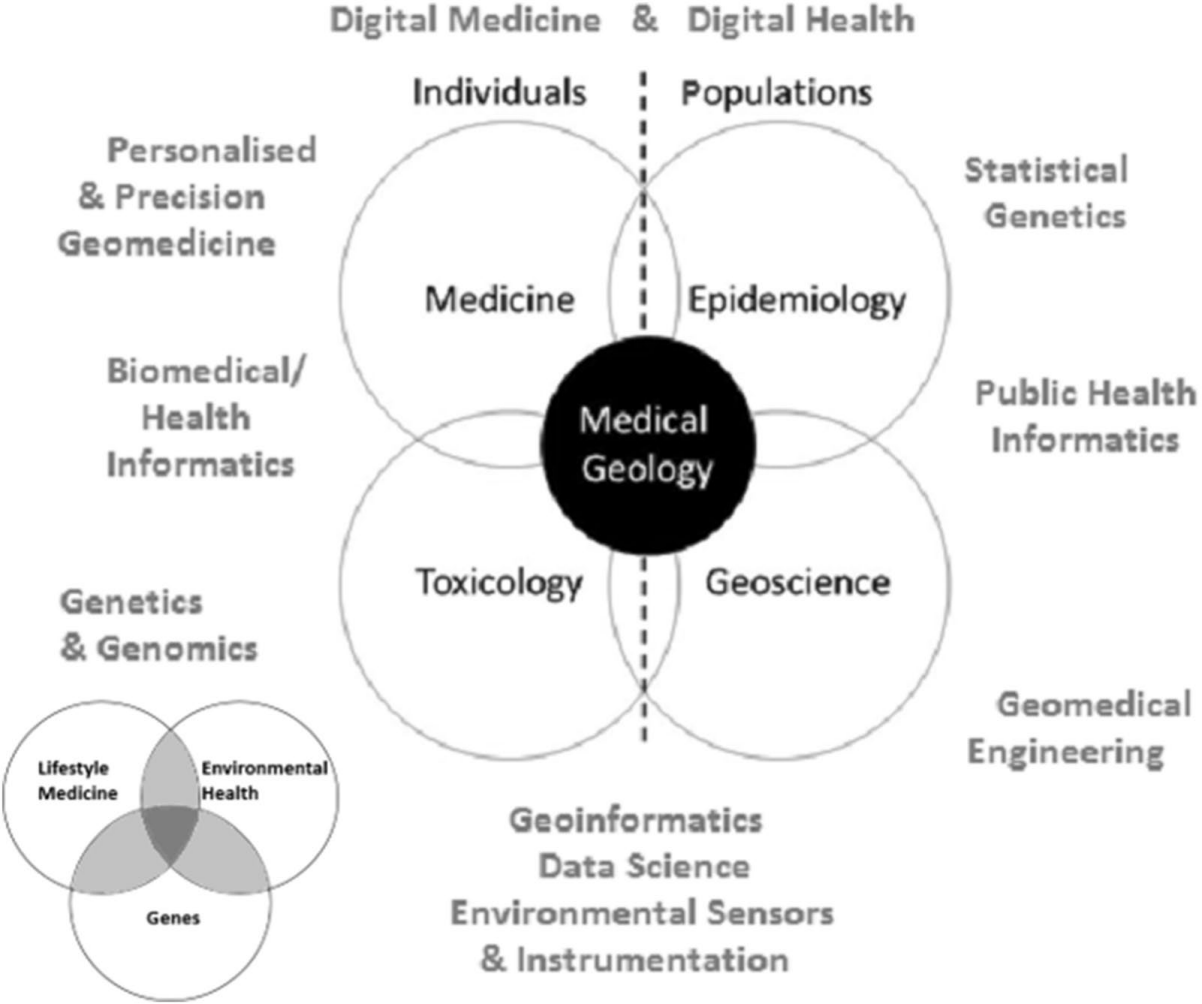
The relationships between the main disciplines required in a modern medical geology study

Many projects carried out in the name of medical geology will involve a multitude of disciplines and expertise in the attempt to find relationships between the environment and health. The paragraphs above introduced the reader to the basic information that must be gathered to build a thorough picture of the intricate steps thought to occur along the exposure pathway, and synthesised in a way that addresses the initial problem. In order to do this, a collective team—crucially those within medicine, epidemiology, toxicology, geoscience and informatics (Fig. 5)—must be willing to speak a language that is translational across the sciences to encourage an integrated understanding of the problem.

Arguably, the spatial component is the one factor that ties in all these disciplines together in the case of medical geology. In a general sense, epidemiology, statistical genetics, geoscience, geomedical engineering and public and environmental health informatics tend to study data in terms of populations, whereas medicine (including personalised and precision geomedicine, and lifestyle medicine), genetics, genomics, toxicology and biomedical/health informatics more likely work on individuals or individual mechanism(s) describing disease.

Digital technologies play important roles in these disciplines. Methods involving the measurement of ‘exposomes’ in the body (via collection of biological samples such as blood, urine, saliva and exhaled breath condensate), to profile subjects’ internal levels of metabolites, metals, serum proteins and persistent organic compounds [32], are complemented by advances in sensor and smartphone technologies to measure (and crowdsource/collate, thinking here of whole populations and regions) multiple geotagged personal exposures to external pollutant levels, as well as other relevant individual/lifestyle factors, such as physical activity and diet [42].

Systems toxicology relies heavily on computational approaches [43, 44]. The widespread use of GIS and related geospatial methods and technologies (e.g., [21, 45, 46]) in medical geology has enabled researchers to map, analyse, and model disease exposure and risk over large geographical areas, and increasingly sophisticated techniques of statistical analysis have made significant advances in the way that disease is monitored and the environment is characterised. Although there are still important challenges in the current approaches used to link disease in humans and animals with environmental exposures (see [47]), digital computational methods involving geostatistics/multivariate statistics (see, for example, [48]), big data analytics and cognitive computing (e.g., IBM Watson [49]) are helping us address the inherent complexities and find interdependent relationships between environmental variables and disease. Readers interested in learning more about medical geology might find it useful to consult [50, 51].

## Conclusions

As we develop more advanced techniques of quantifying and qualifying contaminant exposure, it becomes essential that we fully understand all the stages, internal and external, in the disease pathway, in order to be able to predict and prevent illness due to contaminant exposure. These ultimate goals of disease prediction, prevention and personalised treatment can only be realised through an intensive multiple-disciplinary approach involving the various disciplines mentioned in this article (and most likely some additional ones), to collaborate together and complement each other in additive (multidisciplinary), interactive (interdisciplinary) and holistic (transdisciplinary and cross-disciplinary) manners [52]. A careful consideration and implementation of the promoters of multiple disciplinary teamwork (plus having adequate plans for mitigating any barriers encountered) will secure success [53].
